# Identification of a selective inhibitor of IDH2/R140Q enzyme that induces cellular differentiation in leukemia cells

**DOI:** 10.1186/s12964-020-00536-7

**Published:** 2020-04-03

**Authors:** Jiao Chen, Jie Yang, Qingyun Wei, Ling Weng, Fei Wu, Yun Shi, Xiaolan Cheng, Xueting Cai, Chunping Hu, Peng Cao

**Affiliations:** 1 Affiliated Hospital of Integrated Traditional Chinese and Western Medicine, Nanjing University of Chinese Medicine, 100#, Shizi Street, Hongshan Road, Nanjing, 210028 Jiangsu China; 2 Laboratory of Cellular and Molecular Biology, Jiangsu Province Academy of Traditional Chinese Medicine, Nanjing, 210028 China; 3 Department of Pharmacology, School of Pharmacy, Nanjing University of Chinese Medicine, Nanjing, 210023 China; 4 Engineering Research Center of Modern Preparation Technology of TCM of Ministry of Education, Shanghai University of Traditional Chinese Medicine, Shanghai, 201203 China; 5 Institute for Glycomics, Griffith University, Gold Coast Campus, Queensland, 4222 Australia

**Keywords:** IDH2/R140Q, Selective inhibitor, Acute myeloid leukemia, Molecular simulation, Cell differentiation

## Abstract

**Background:**

IDH2/R140Q mutation is frequently detected in acute myeloid leukemia (AML). It contributes to leukemia via accumulation of oncometabolite D-2-HG. Therefore, mutant IDH2 is a promising target for AML. Discovery of IDH2 mutant inhibitors is in urgent need for AML therapy.

**Methods:**

Structure-based in silico screening and enzymatic assays were used to identify IDH2/R140Q inhibitors. Molecular docking, mutant structure building and molecular dynamics simulations were applied to investigate the inhibitory mechanism and selectivity of CP-17 on IDH2/R140Q. TF-1 cells overexpressed IDH2/R140Q mutant were used to study the effects of CP-17 on cellular proliferation and differentiation, the wild-type TF-1 cells were used as control. The intracellular D-2-HG production was measured by LC-MS. The histone methylation was evaluated with specific antibodies by western blot.

**Results:**

CP-17, a heterocyclic urea amide compound, was identified as a potent inhibitor of IDH2/R140Q mutant by in silico screening and enzymatic assay. It exhibits excellent inhibitory activity with IC_50_ of 40.75 nM against IDH2/R140Q. More importantly, it shows poor activity against the wild-type IDH1/2, resulting in a high selectivity of over 55 folds, a dramatic improvement over previously developed inhibitors such as AGI-6780 and Enasidenib. Molecular simulations suggested that CP-17 binds to IDH2/R140Q at the allosteric site within the dimer interface through extensive polar and hydrophobic interactions, locking the enzyme active sites in open conformations with abolished activity to produce D-2-HG. Cellular assay results demonstrated that CP-17 inhibits intracellular D-2-HG production and suppresses the proliferation of TF-1 erythroleukemia cells carrying IDH2/R140Q mutant. Further, CP-17 also restores the EPO-induced differentiation that is blocked by the mutation and decreases hypermethylation of histone in the TF-1(IDH2/R140Q) cells.

**Conclusions:**

These results indicate that CP-17 can serve as a lead compound for the development of inhibitory drugs against AML with IDH2/R140Q mutant.

Video abstract.

## Background

Mutations in the metabolic genes contribute to the initiation, development and maintenance of tumors [1]. Isocitrate dehydrogenase 2 (IDH2) is a vital metabolic enzyme involved in the Krebs cycle, catalyzing the production of α-ketoglutarate (α-KG) from isocitrate. Point mutations of two arginine residues, R140Q and R172K in the active site of IDH2, can drive the development of leukemia and glioma in vivo [2–5]. These cancer-associated IDH2 mutants gain neomorphic activity that converts α-KG to a reduced product (D)-2-hydroxyglutarate (D-2-HG), as high concentrations of D-2-HG can be detected in glioma and acute myeloid leukemia (AML) patients harboring these mutations [5, 6]. It has been shown that accumulation of D-2-HG competitively inhibits α-KG-dependent dioxygenases, such as histone and DNA demethylases that play critical roles in the regulation of the cellular epigenetic state [7, 8]. Epigenetic dysregulation has been proved to block hematopoietic differentiation [9–13]. Among these mutations, approximately 10% of AML patients carry R140Q mutation in the *IDH2* gene, making it a promising target for the therapy of AML [14, 15].

Currently, only a handful of inhibitors of IDH2/R140Q have been reported, namely AGI-6780 [11], Enasidenib (AG-221) [16, 17], and a class of pyridine derivatives [18]. AGI-6780 is a preclinical inhibitor of IDH2/R140Q enzyme, which can lower D-2-HG concentration and induce cellular differentiation, providing in vitro evidence that the mutation-induced epigenetic changes can be reversed by the inhibition of IDH2/R140Q. Enasidenib mesylate is an oral and first-in-class inhibitor of the mutant IDH2, which has been approved by the Food and Drug Administration (FDA) in 2017 for treating adult patients with relapsed/refractory AML carrying mutant IDH2. Although patients responding to Enasidenib show evidence of hematopoietic recovery, 11.7% of these patients develop differentiation syndrome, which is a lethal adverse effect of Enasidenib if not adequately treated [19, 20]. Additionally, therapeutic resistance caused by the emergence of second-site IDH2 mutations occurs after a certain period of treatment with Enasidenib [21]. Thus, novel inhibitors of IDH2 mutants with improved selectivity and safety profiles are desired.

In this study, we identified a heterocyclic urea amide compound CP-17 as an inhibitor of the IDH2/R140Q mutant from virtual screening. It exhibits excellent inhibitory activity with an IC_50_ value of 40.75 nM. Further, CP-17 exhibits poor activity against the wild-type IDH2 (IDH2/WT), showing a high selectivity of more than 55 folds, a dramatic improvement over AGI-6780 and Enasidenib. Molecular simulations suggested that CP-17 binds to an allosteric site located within the interface of IDH2/R140Q homodimer, stabilizing an open conformation of the active site. The binding of CP-17 was shown to disrupt the transition of the active site to a closed conformation, thus preventing the catalytic reaction. Moreover, CP-17 exhibits robust D-2-HG suppression activity in TF-1 (IDH2/R140Q) cells and reverses the cellular differentiation block induced by the R140Q mutation. Herein, we provide details of the discovery of CP-17 and its in vitro activity against the mutant IDH enzymes by computational and experimental methods. The in vitro efficacy of CP-17 for IDH2/R140Q mutated TF-1 cells has also been demonstrated.

## Materials and methods

### Reagents, enzymes, and antibodies

Compounds of CP-17 and AGI-6780 were purchased from TargetMol (Shanghai, China) and MedChemExpress (Shanghai, China) respectively. The human recombinant C-terminal FLAG-tag IDH enzymes including IDH2/R140Q, IDH2/WT, IDH1/R132H and IDH1/WT homodimer were purchased from BPS Bioscience (San Diego, USA). Antibodies of Hemoglobin γ (HBG) (catalog number: 39386S), H3K9me3 (catalog number: 5327S), H3K27me3 (catalog number: 9733S), H3K4me3 (catalog number: 9725S), and H3 (catalog number: 4499S) were purchased from Cell Signaling Technology (Massachusetts, USA). Recombinant human granulocyte-macrophage colony-stimulating factor (GM-CSF) and erythropoietin (EPO) were obtained from Peprotech (New jersey, USA). NADPH, NADP, α-KG, isocitrate, D-2-HG acid disodium, DMSO, diaphorase and resazurin were purchased from Sigma-Aldrich (Missouri, USA).

### Structure-based in silico screening of IDH2/R140Q inhibitors

Structure-based in silico screening of IDH2/R140Q inhibitors was performed using Glide in Schrödinger 2015–3 software. The crystal structure of IDH2/R140Q in complex with an allosteric inhibitor (PDB ID: 4JA8) was obtained from the RCSB protein data bank (http://www.rcsb.org/). Residues within 15 Å around the allosteric inhibitor in the crystal structure were defined as the binding site at which the docking grids were created. The library of ChemDiv with 1.26 million compounds was prepared in LigPrep panel. The compounds were protonated at pH 7.0 ± 2.0 and the low-energy conformations were generated using OPLS3 force field. Epik was adopted to generate the ionization states. Three stages of virtual screening (HTVS: High-throughput Virtual Screening mode; SP: Standard-Precision mode; and XP: Extra-Precision mode) with increasing accuracy and computational cost were carried out. At each stage, only the top 10% scoring compounds were selected to advance to the next stage. After the extra-precision screening, the structures and binding interaction modes of the retained compounds were analyzed and finally, 31 compounds were purchased for bioassay.

### Enzymatic assay of IDH inhibitors

The compounds were dissolved to a concentration of 20 mM in pure DMSO. Diaphorase/resazurin coupled NADPH deletion or production assays were used to measure the activity of IDH mutants or wild-type IDH (IDH/WT) enzymes [11]. A mixture of IDH2/R140Q enzyme (0.60 μg/mL) and NADPH (30 μM) was prepared in 40 μL assay buffer that consists of 10 mM MgCl_2,_ 150 mM NaCl, 50 mM K_2_HPO_4_/KH_2_PO_4_ (pH 7.5), 2 mM β-mercapoethanol, 0.05% BSA and 10% glycerol. Afterwards, 20 μL of the gradient concentrations of compound dilution were added to this mixture and incubated at 25 °C for 1 h or 16 h. Then, 20 μL of 4X assay buffer containing 16 mM α-KG was added to initiate the enzymatic reaction, which was allowed to proceed for 1 h at 25 °C. Likewise, the IDH2/WT enzyme (0.12 μg/mL) was mixed with NADP (50 μM) in 40 μL assay buffer, after which compound dilutions were added and incubated as the IDH2/R140Q enzyme assay. Further, 20 μL of 4X assay buffer containing 0.6 mM isocitrate was used for enzymatic reaction. Finally, for both the IDH2/R140Q and IDH2/WT enzymes assays, 40 μL of 3X detection buffer (assay buffer with 45 μg/mL diaphorase and 90 μM resazurin) was added to stop the reaction and incubated for 10 min at room temperature. Then, 40 μL of the resulting mixture was added into a 384-well dark plate in duplicate and the absorbance was measured using Envision (Ex/Em = 544/590 nm).

Analogously, a mixture of the IDH1/R132H enzyme (0.2 μg/mL) and NADPH (30 μM) was prepared in the assay buffer [10 mM MgCl_2_, 150 mM NaCl, 20 mM Tris-Cl (pH 7.5), 0.05% BSA, and 2 mM β-mercaptoethanol], after which compound dilutions were added and incubated for 16 h at 25 °C. The activity was measured using the same method as for IDH2/R140Q.

A mixture of the IDH1/WT (0.08 μg/mL) and NADP (50 μM) was prepared in the assay buffer [10 mM MgCl_2_, 150 mM NaCl, 20 mM Tris (pH 7.5), 2 mM β-mercaptoethanol, and 0.05% BSA], after which compound dilutions were added and incubated for 16 h at 25 °C. Other operations were referred to the assay of IDH2/WT.

### Molecular dynamics (MD) simulations

The structure of IDH2/WT was modeled in Discovery Studio 4.1 software using IDH2/R140Q (PDB ID: 4JA8) as the template. CP-17 was docked into IDH2/WT in Glide. The complexed structures of IDH2/R140Q or IDH2/WT binding with the inhibitor CP-17 and IDH2/R140Q with the substrate α-KG (PDB ID: 5I95) were prepared in the Schrödinger 2015–3 software. MD simulations were conducted in Amber 12 in reference to our previous study [22]. The structures of CP-17 and α-KG were optimized at HF/6-31G* level in Gaussian 09 program, and then RESP charges and GAFF force field were assigned for the optimized structures. The NADPH and calcium ion parameters were obtained from the Amber parameter database (http://research.bmh.manchester.ac.uk/bryce/amber). Amber ff12SB force field was assigned for the protein. The systems were solvated in a periodic box of TIP3P water molecules that extend 10.0 Å from the protein atoms. Cl^−^ counterions were added to neutralize the systems.

Energy minimization (5000 steps for the water molecules followed by 5000 steps for the whole system) were performed before MD simulations. Then the systems were simulated at constant temperature (300 K) and pressure (1.0 atm) controlled by the Langevin algorithm [23] with a time step of 1.5 fs. Electrostatic interactions were calculated using the PME method [24], and the non-bonded cutoff was set to 10.0 Å. The hydrogen bonds were constrained by SHAKE algorithm [25]. Each system was equilibrated for 500 ps and then 200 ns production simulations were conducted with a time step of 2 fs. The data were analyzed in cpptraj module and the structures were visualized in PyMOL 1.7.4.

### Cell culture

TF-1(WT) and TF-1(IDH2/R140Q) cells were acquired from the American Type Culture Collection (ATCC) (Manassas, USA) in January 2018. TF-1 and TF-1(IDH2/R140Q) cells were cultured in RPMI-1640 (ATCC30-2001), with 10% FBS (ATCC30-2020) and 2 ng/mL human GM-CSF. All cells were cultured under 5% CO_2_ at 37 °C. The last mycoplasma test of the two cell lines was on December 2018 and no mycoplasma contamination was found.

### Differentiation assay

TF-1(WT) and TF-1(IDH2/R140Q) cells were washed three times by PBS to remove GM-CSF and treated with CP-17 (1 or 3 μM) and 5 ng/mL EPO for 7 days.

### GM-CSF-independent proliferation assay

The cell proliferation was detected by CellTiter 96® AQueous One Solution Cell Proliferation Assay Kit (Promega) according to the protocol. Briefly, TF-1(WT) and TF-1(IDH2/R140Q) cells were cultured in complete growth medium that is supplemented with 2 ng/mL GM-CSF. Prior to the proliferation assay, the cells were washed three times with PBS to eliminate GM-CSF. These cells were transferred to 96 well plates (5000 cells per well), with each well containing 100 μL growth medium without GM-CSF, and treated with CP-17 (1 or 3 μM) for 7 days. Then 20 μL per well of CellTiter 96® AQueous One Solution Reagent were added to each well. The plate was incubated at 37 °C for 4 h in a humidified, 5% CO2 atmosphere, and then the absorbance at 490 nm was recorded using a 96-well plate reader. Three independent repetitive experiments were carried out and analyzed by Graphpad Prism 5.0. The mean and s.d. values were calculated and showed in the form of histogram. T-test was used to compare the difference significance with control group and the data meet the assumption of the test.

### D-2-HG assay

TF-1(IDH2R140Q) cells were seeded in a 12-well plate at a density of 5 × 10^4^/mL. The test compound was added in different concentration gradients (3000, 1000, 333.33, 111.11, 37.04, 12.35, 4.12, and 1.37 nM). The cells were collected after 3 days of treatment. The cell pellet was suspended in 100 μL 80% aqueous methanol solution, containing 1 μg/mL Phenacetin as the internal standard. The cell lysate was centrifuged at 13,000 rpm for 10 min. The precipitate was removed at 4 °C and stored in an − 80 °C refrigerator. Before injection, the cell lysate was centrifuged again at 13,000 rpm for 10 min. The supernatant was transferred into the liquid phase cannula for injection. The test instrument was a Q Exactive™ Plus-Orbitrap™ MS, Thermo Scientific benchtop quadrupole-orbitrap high resolution mass spectrometer. The column used was Waters™ ACQUITY™ UPLC HSS-T3–1.8 μm, 21 mm × 100 mm (part No. 186003539). The mobile phase was: Phase A, 0.1% aqueous formic acid (chromatographically pure); and Phase B, acetonitrile (chromatographically pure). The gradients were set as 1% B for 1.5 min → 70% B for 0.5 min → 1% B for 1.5 min → 0% B. The column was re-equilibrated for 2 min between injections. A temperature of 40 °C was set for the column with a flow rate of 0.2 mL/min. The selected reaction monitoring was used for data acquisition with Xcalibur software. D-2-HG and Phenacetin were monitored by mass transitions of m/z 147 → m/z 129 and m/z 178 → m/z 147, respectively. Calibration and integration of peaks were performed using GraphPad Prism 5.0 software.

### Histone methylation assay

TF-1(WT) and TF-1(IDH2/R140Q) cells were treated with CP-17 (1 or 3 μM) in the presence of recombinant human GM-CSF for 7 days and then lysed and evaluated for the expression of H3K9me3, H3K4me3, H3K27me3, and H3 with specific antibodies by western blot. The total amount of histone H3 was used as the loading control. Three independent repetitive experiments were carried out.

## Results

### Identification of CP-17 as an allosteric inhibitor of IDH2/R140Q

**Fig. 1:**
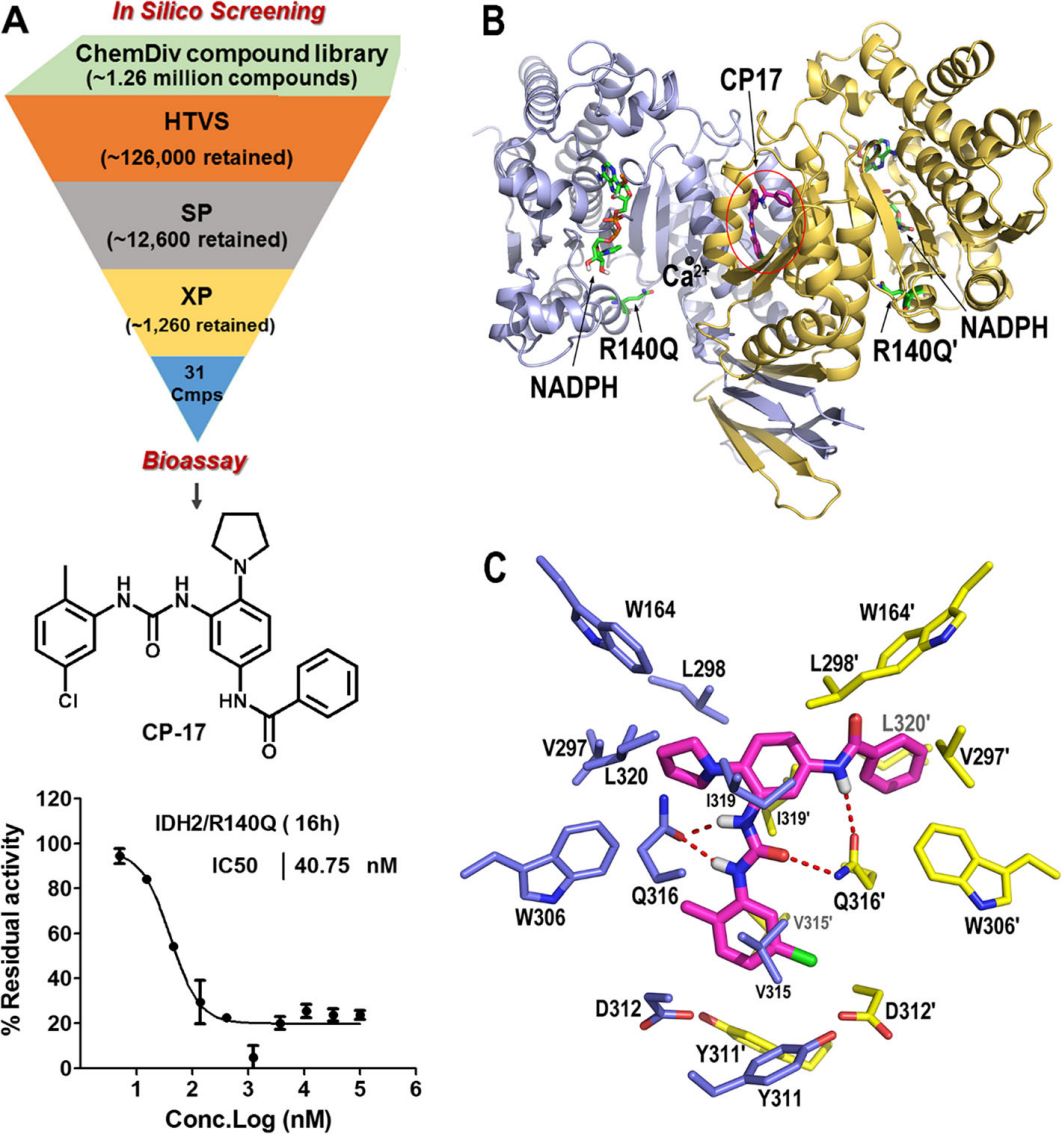
Identification of CP-17 as an inhibitor of IDH2/R140Q. **a** Structure-based in silico screening of a small molecule library (ChemDiv) for compounds that bind to the allosteric site of IDH2/R140Q identifies a hit compound CP-17 with IC_50_ of 40.75 nM. HTVS: High-throughput Virtual Screening mode; SP: Standard-Precision mode; XP: Extra-Precision mode. **b** Structure of CP-17 binding at the allosteric site of IDH2/R140Q. The protein was represented as cartoon (colored in light blue and yellow for each monomer), the carbon atoms of CP-17 as magenta sticks, NADPH and Q140 as green sticks, and the Ca^2+^ as black spheres. **c** Molecular interactions of CP-17 binding at the IDH2/R140Q dimer interface (light blue and yellow for carbon atoms of adjacent monomer residues). The hydrogen bonds between CP-17 and Q316 residue were labeled with red dotted lines

Molecular simulation is an important means of drug discovery [26]. Inspired by the important role of oncogenic IDH2 mutations, we employed a structure-based in silico screening method to discover inhibitors of the most prevalent IDH2 mutation−IDH2/R140Q in AML using Schrödinger 2015–3 software. Following a cascade of three screening stages (HTVS, SP and XP), dozens of candidate compounds from ChemDiv library were identified and purchased for enzyme inhibitory assay (Fig. 1a). Although the IDH2 R140Q/WT heterodimer might be a more relevant biological target, the R140Q homodimer was used as a target in this assay due to the fact that the homogeneous assay measuring the amount of NADPH is not suitable when both mutant and wild-type reactions occur simultaneously. Enzyme inhibitory assays identified CP-17 [*N*-(3-(3-(5-chloro-2-methylphenyl)ureido)-4-(pyrrolidin-1-yl)phenyl)benzamide] as a potent inhibitor of IDH2/R140Q (Fig. 1a). It inhibited the activity of IDH2/R140Q homodimer in a dose-dependent manner with an IC_50_ value of 40.75 nM (16 h) in vitro (Fig. 1a, Table 1).

**Table 1:** In vitro inhibition of the inhibitors with homodimeric IDH enzymes

Enzymes assayed	Incubation time (hours)	IC_50_ (nM)		
		CP-17	AGI-6780	Enasidenib^c^
IDH2/R140Q^a^	1	99.88	16.39	320
	16	40.75	12.83	100
IDH2/WT^b^	16	2267	99.95	1800
**Selectivity**	16	**55.6**	**7.8**	**18**
IDH1/R132H^a^	16	> 5000		
IDH1/WT^b^	16	> 5000		

^a^ IC_50_ for α-KG reduction; ^b^ IC_50_ for isocitrate to α-KG; ^c^ IC_50_ reported from reference [16]

IDH2 is an NADPH-dependent and divalent cation-dependent enzyme. Its catalytic pocket consists of binding sites for the substrate, the cofactor NADPH, as well as a divalent metal cation. The mutated residue R140Q is adjacent to the catalytic active site (Fig. 1b). Molecular docking study suggests that CP-17 binds to the allosteric site at the homodimer interface, which is comprised of four helices (formed by the residues 290–299 and 310–322 from each monomer). Owing to the distance between the allosteric site and the substrate-binding site, CP-17 shows no direct interactions with Q140 (Fig. 1b). The binding of CP-17 involves multiple hydrogen bonds and hydrophobic interactions. The nitrogen atoms of the urea group and amide group on CP-17 donate three hydrogen bonds to the amide oxygen of Q316 site chain (locating at the divalent cation binding helix) from both monomers, whereas the carbonyl oxygen of the urea group accepts one hydrogen bond from the amide nitrogen of Q316 side chain on one monomer. Through its pyrrolidinyl and phenyl groups, CP-17 is also involved in hydrophobic interactions with V297, L298, V315, I319, L320, and Y311, contributing to its inhibitory potency (Fig. 1c). The predominant hydrophobic site indicates that large polar substituents on the benzene rings of the inhibitor are less favorable.

### CP-17 is a selective and time-dependent binder of IDH2/R140Q

CP-17 is a time-dependent and slow tight binder of IDH2/R140Q enzyme with IC_50_ of 99.88 nM at 1 h incubation and 40.75 nM at 16 h incubation for inhibiting D-2-HG production (Table 1, Supplementary Fig. 1). Although the inhibitory activity is a little worse than the reported inhibitor AGI-6780 (IC_50_: 12.83 nM at 16 h incubation), CP-17 exhibits excellent selectivity for IDH2/R140Q homodimer over IDH2/WT, IDH1/R132H mutant, and IDH1/WT enzymes (Table 1, Supplementary Fig. 1), as it is 55.6 times more potent for IDH2/R140Q than for IDH2/WT. This selectivity was much higher than AGI-6780 (7.8 folds) and Enasidenib (18 folds). The chemical structures of CP-17 and AGI-6780 are similar except that AGI-6780 is a heterocyclic urea sulfonamide compound [11] while CP-17 is heterocyclic urea amide. The substitution of amide with sulfonamide might contribute to the selectivity of CP-17. A recent report suggests that the −CF_3_ group of AGI-6780 and Enasidenib increases the binding affinity of the inhibitor with the allosteric site of IDH2/R140Q [27]. Therefore, we speculate that replacing the –Cl with −CF3 group will increase the inhibitory potency of CP-17.

### CP-17 allosterically regulates the conformations of the enzyme active sites

**Fig. 2:**
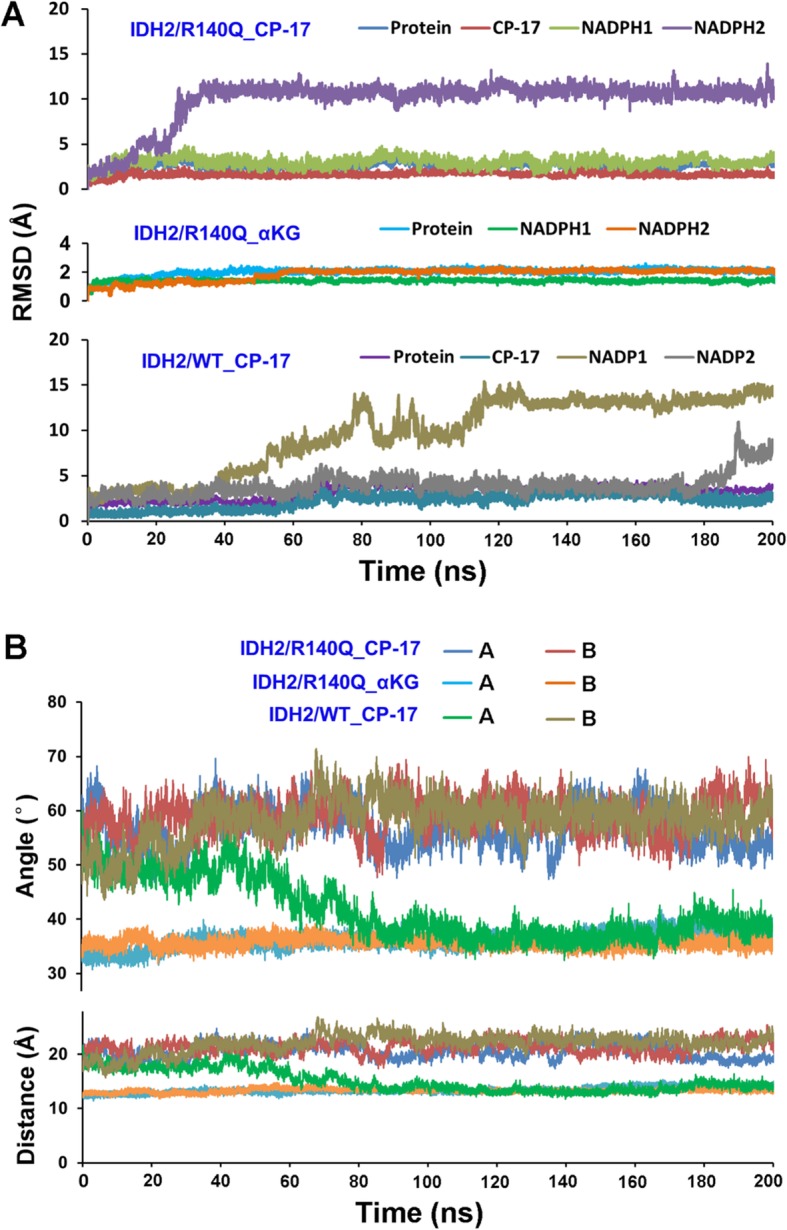
Molecular dynamics (MD) simulations of IDH2/R140Q_CP-17, IDH2/R140Q_α-KG and IDH2/WT_CP-17 systems. **a** Evolution of RMSDs during 200 ns MD simulations. **b** Evolution of the Ile116-Leu289′ distances and the Ile116-Phe148-Leu289′ angles (measured on Cα atoms) in monomers A and B during MD simulations

As shown by molecular docking, CP-17 binds to the allosteric site of IDH2/R140Q enclosed with the homodimer interface. To examine its inhibition mechanism of the neomorphic activity of IDH2/R140Q, 200 ns-long MD simulation of IDH2/R140Q in complex with CP-17 (IDH2/R140Q_CP-17) was performed. The system IDH2/R140Q_α-KG was also simulated as control. The cofactor NADPH was present in both systems. During simulations, the conformation of protein in both systems reached equilibrium after about 50 ns, as indicated by the stabilization of their respective root-mean-square deviation (RMSD) values; however, the behaviors of NADPH in the two systems were distinct (Fig. 2a). The RMSDs of NADPH in IDH2/R140Q_CP-17 system ranged from 0.66 to 18.32 Å, suggesting that the NADPH underwent considerable conformational changes. In comparison, its RMSDs in the system of IDH2/R140Q_α-KG experienced less fluctuation, ranging from 0.35 to 2.45 Å, indicating better stability of NADPH conformation (Fig. 2a). Similar effects were observed in our previous study [22]. To verify changes in NADPH binding to IDH2/R140Q, the binding free energy was calculated in the presence or absence of CP-17. In the IDH2/R140Q_CP-17 system, NADPH binds to the enzyme with binding free energies of − 753.79 and − 830.36 kcal/mol in the two monomers, respectively, much lower than that in the IDH2/R140Q_α-KG system (Table 2). These results indicated that the binding of allosteric inhibitors could induce conformational changes of the NADPH binding site in IDH2/R140Q and affect the stability of NADPH interactions with the enzyme, thereby undermining the catalytic activity of the NADPH-dependent enzyme. Indeed, a higher inhibitory activity is associated with a weaker interaction of NADPH to the enzyme, as compared with AGI-6780 (NADPH binds to the IDH2/R140Q_AGI6780 system with binding free energies of − 509.75 and − 646.63 kcal/mol in the two monomers, respectively) [22].

**Table 2:** The calculated binding free energy of NADPHs with IDH2/R140Q in different complexes

NADPH	Binding free energy (kcal/mol)	
	IDH2/R140Q_CP-17	IDH2/R140Q_α-KG
NADPH^A^	−753.79	− 1418.89
NADPH^B^	−830.36	− 1382.28

**Fig. 3:**
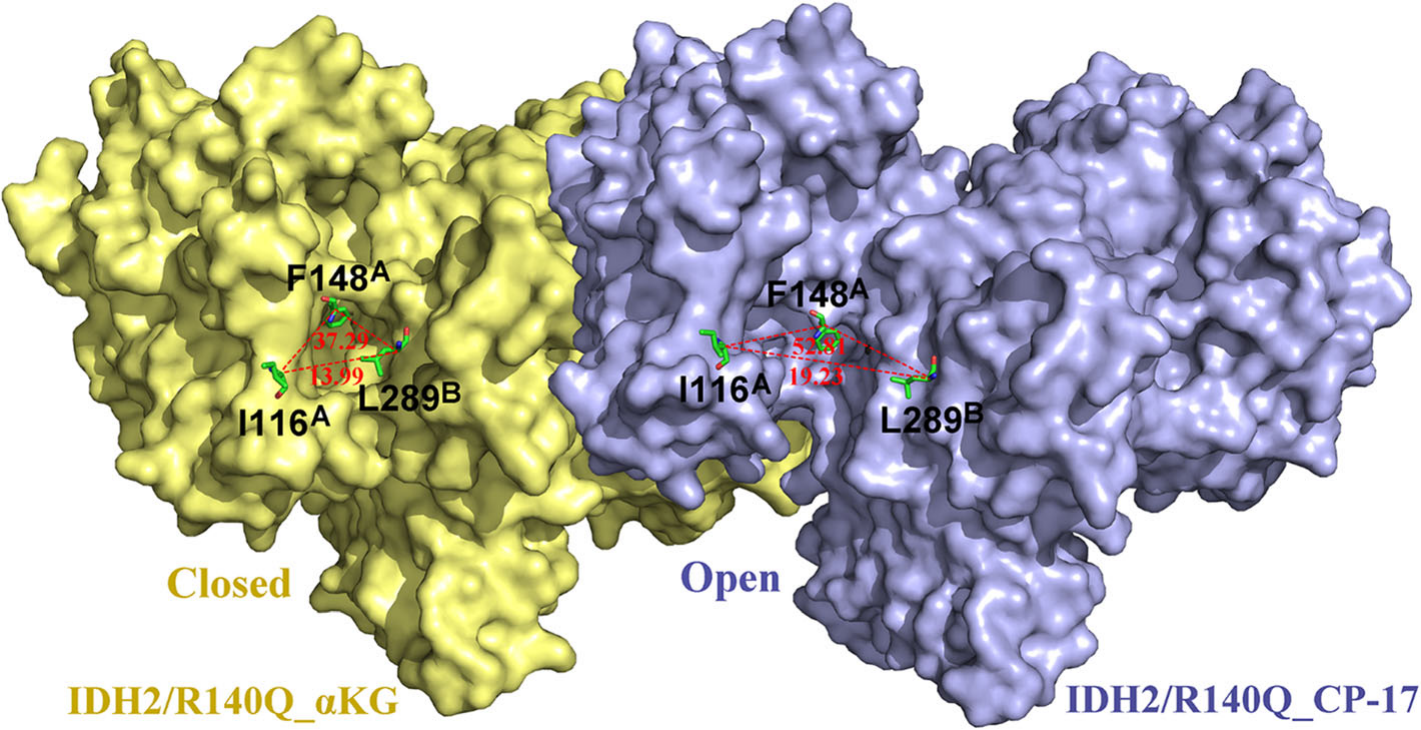
Representation of the surface maps of IDH2/R140Q_α-KG and IDH2/R140Q_CP-17. The α-KG-bound IDH2/R140Q structure with the catalytic site in the closed active conformation (left) versus the CP-17 bound IDH2/R140Q structure with the catalytic site in an inactive open conformation (right)

MD simulations suggested that the width of the catalytic pocket entrance in IDH2/R140Q_CP-17 (defined as the distance between I116-L289’ and the angle of I116-F148-L289’) is much broader than in IDH2/R140Q_α-KG. The evolving distances and angles in both the systems during MD simulations are shown in Fig. 2b. In the two monomers of IDH2/R140Q_CP-17 system, the average I116-L289′ distances are 20.50 Å and 21.49 Å, respectively, while the average I116-F148-L289′ angles are 56.68° and 59.41°, respectively, during the 100–200 ns simulation period (Table 3). These values are much larger than those in the IDH2/R140Q_α-KG system (Fig. 2b, Table 3), suggesting much wider catalytic pockets in the presence of CP-17 instead of α-KG. Surface maps illustrated that CP-17 allosterically stabilizes the active site of IDH2/R140Q in an open conformation (Fig. 3). As a closed conformation upon α-KG binding is essential for the catalytic activity of IDH2/R140Q [28], such an open conformation potentially prevents the conversion of α-KG to D-2-HG. In contrast, the inhibitor-free IDH2/R140Q_α-KG complex, known to have neomorphic activity, adopts a compact closed conformation for its active site. Additionally, the two monomers of IDH2/R140Q exhibit different distances and angles for the entrance of the substrate-binding site (Table 3). In the allosteric inhibitor binding systems, one monomer of IDH2/R140Q_CP-17 adopts a more open conformation than the other.

**Table 3:** The average distances of Ile116-Leu289’ and angles of Ile116-Phe148-Leu289’ between Cα atoms calculated from the 100–200 ns MD simulation trajectory

Systems	Distance		Angle	
	I116^A^-L289^B^ (Å)	I116^B^-L289^A^ (Å)	I116^A^-F148^A^-L289^B^ (°)	I116^B^-F148^B^-L289^A^ (°)
IDH2/R140Q_CP-17	20.50 ± 1.38^*^	21.49 ± 1.18^*^	56.68 ± 3.42^*^	59.41 ± 3.15^*^
IDH2/R140Q_α-KG	13.78 ± 0.56	13.32 ± 0.27	37.01 ± 1.36	35.52 ± 0.82
IDH2/WT_CP-17	13.61 ± 0.69^#^	22.56 ± 0.86^*^	37.59 ± 1.77^#^	59.25 ± 2.27^*^

Compared with IDH2/R140Q_α-KG, ^*^P < 0.001

Compared with IDH2/R140Q_CP-17, ^#^*P* < 0.001

**Fig. 4:**
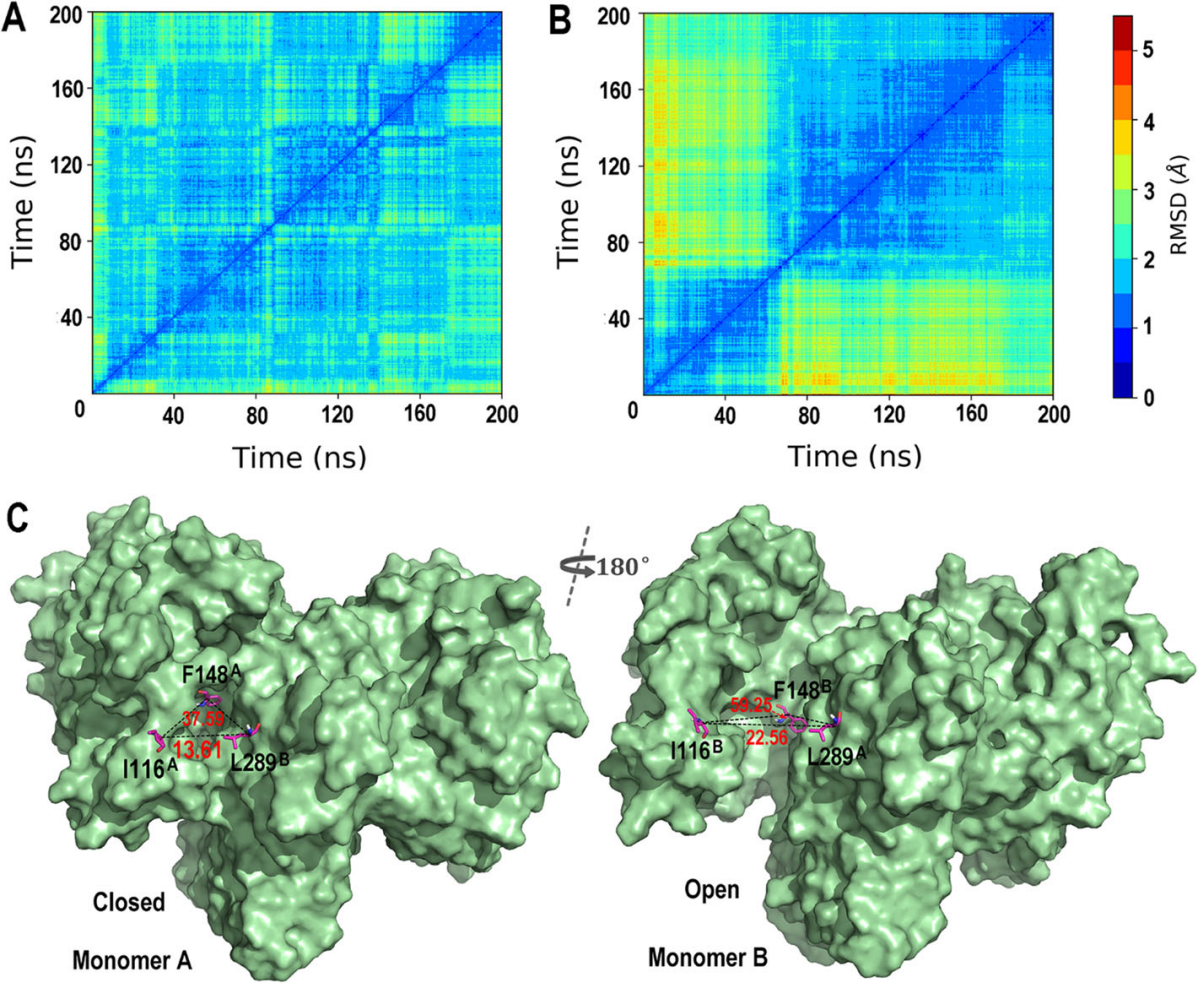
Conformational changes of IDH2/WT bound with CP-17 after MD simulation. RMSD matrices of IDH2/R140Q_CP-17 **a** and IDH2/WT_CP-17 **b** systems during MD simulation. **c** Representation of surface maps of CP-17 bound IDH2/WT structure, with the catalytic sites of monomer A in an active closed conformation (left) and monomer B in an inactive open conformation (right)

CP-17 exhibits excellent selectivity with 55.6 times more potent activity against IDH2/R140Q than IDH2/WT, the mechanistic basis of which remains unclear. To investigate this question, the structure of IDH2/WT was modeled based on IDH2/R140Q (PDB ID: 4JA8) and docked with CP-17, then 200 ns MD simulation of the IDH2/WT_CP-17 complex was performed. Like NADPH in IDH2/R140Q, the RMSDs of the cofactor NADP^+^ in IDH2/WT fluctuated significantly during simulation (Fig. 2a). The RMSD matrices of the protein suggested that the structure of IDH2/WT experienced marked conformational changes, mainly two clusters (Fig. 4a, b). The allosteric interaction mode of CP-17 with IDH2/WT was similar to that with IDH2/R140Q. Distinctively, the substrate-binding pocket of monomer A gradually closed from the open conformation compared with the structure of IDH2/R140Q_CP-17, with the average distance of I116^A^-L289^B^ 13.61 Å and angle of I116^A^-F148^A^-L289^B^ 37.59° in the last 100 ns trajectory, which is approximate to the values of that in IDH2/R140Q_α-KG (Table 3, Fig. 2b). Nevertheless, the width of the catalytic pocket entrance in monomer B changed little during the simulation. CP-17 inhibits IDH2/WT with IC_50_ of 2267 nM. The simulation results demonstrated that CP-17 locks one monomer of the enzyme in an inactive open conformation and the other in an active closed conformation (Fig. 4c). These conformation states decreased the enzymatic activity of IDH2/WT, however, compared with IDH2/R140Q inhibited by CP-17 (both monomers are locked in open conformations), IDH2/WT is more active when treated with the same concentrations of CP-17 (Supplementary Fig. 1). This helps understanding the selectivity of CP-17 for IDH2/R140Q rather than IDH2/WT.

### CP-17 inhibits the proliferation of TF-1(IDH2/R140Q) cells

**Fig. 5:**
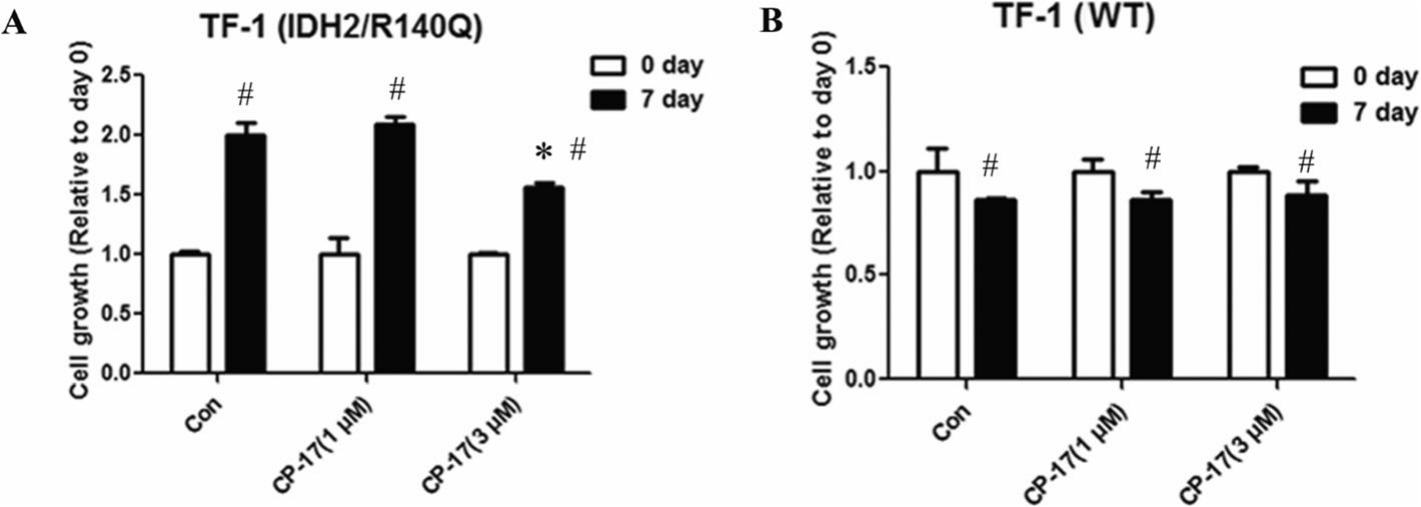
CP-17 treatment reversed GM-CSF-independent growth induced by IDH2/R140Q. IDH2/R140Q expression confers GM-CSF independent growth of the TF-1 cells. Proliferation of TF-1(IDH2/R140Q) (**a**) and TF-1(WT) (**b**) cells with the treatment of CP-17. (* *P* < 0.01 vs 7 day of Control, ^#^*P* < 0.01 vs 0 day of Control). The variance is similar between the groups that are being statistically compared

To investigate the biological effects of CP-17, cellular assays were performed with TF-1 erythroleukemia cells carrying IDH2/R140Q mutant protein. As shown in Fig. 5, TF-1(IDH2/R140Q) cells can grow in the absence of GM-CSF, while the wild-type TF-1 (TF-1(WT)) cell growth was GM-CSF-dependent. Cellular proliferation assay suggested that treatment with 3 μM CP-17 significantly inhibited the GM-CSF-independent growth of TF-1(IDH2/R140Q) cells but had no obvious effect on the proliferation of TF-1(WT) cells (Fig. 5).

### CP-17 restores the EPO-induced differentiation of TF-1(IDH2/R140Q) cells

**Fig. 6:**
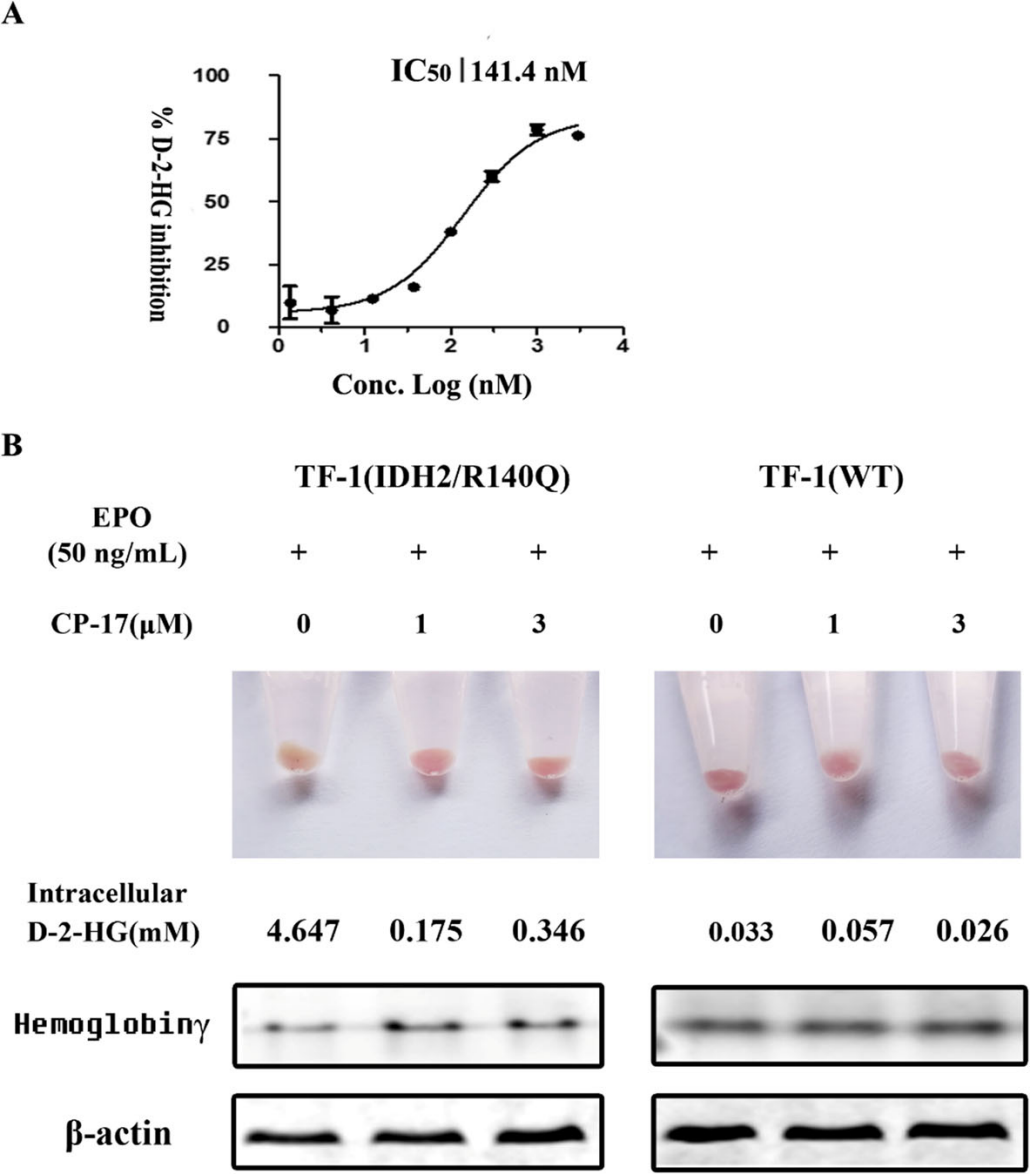
CP-17 reversed the TF-1(IDH2/R140Q) differentiation block. **a** IC_50_ value of intracellular D-2-HG inhibition by CP-17 treatment in TF-1(IDH2/R140Q) cells. **b** TF-1(WT) or TF-1(IDH2/R140Q) cells were induced with 50 ng/mL EPO to differentiate for 7 days in the presence of 0, 1 μM, and 3 μM of CP-17. After then cells were collected, and color change induced by hemoglobin γ expression was photographed. D-2-HG level and hemoglobin γ protein expression were detected with LC-MS and western blot respectively

Similar to human tumor cells, TF-1(IDH2/R140Q) cells were known to produce high levels of D-2-HG [11]. CP-17 showed a dose-dependent inhibition against intracellular D-2-HG production by TF-1(IDH2/R140Q) cells, with an IC_50_ of 141.4 nM (Fig. 6a). Treatment of TF-1 (WT) cells with EPO-induced differentiation were associated with a red color change and increasing hemoglobin γ expression (Fig. 6b). Such differentiation induced by EPO was not detected in TF-1(IDH2/R140Q) cells with increased intracellular D-2-HG level, confirming that IDH2/R140Q mutation blocked differentiation induced by EPO. However, treatment of TF-1(IDH2/R140Q) cells with 1 μM CP-17 decreased intracellular D-2-HG level and restored the EPO-induced color change and hemoglobin γ expression, which were indicative of cellular differentiation.

### CP-17 decreases hypermethylation of histone in TF-1(IDH2/R140Q) cells

**Fig. 7:**
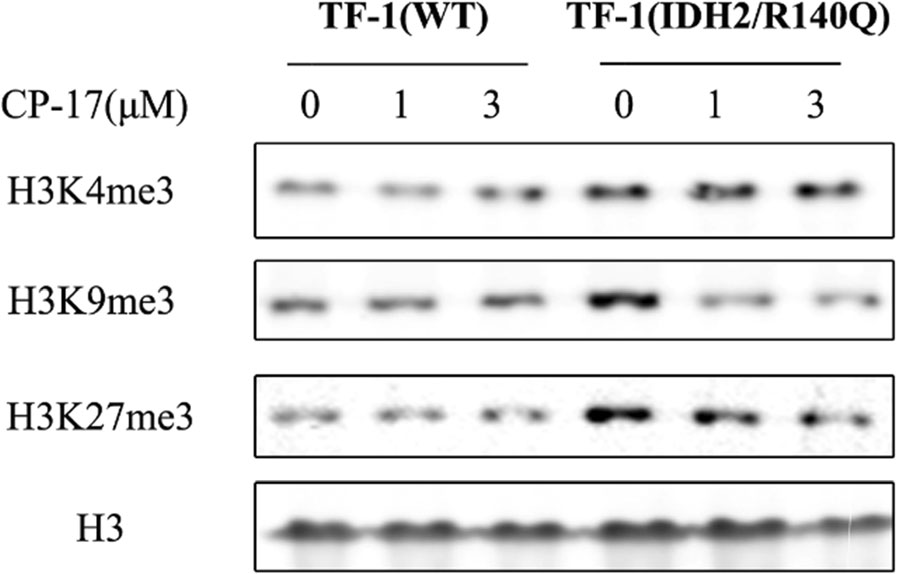
CP-17 decreased high level of histone methylation in TF-1(IDH2/R140Q) cells. TF-1(IDH2/R140Q) and TF-1(WT) cells were treated with CP-17 (1 μM, 3 μM) for 7 days, after which expression levels of H3K4Me3, H3K9Me3, H3K27Me3 and H3 were assessed by western blot with specific antibodies. The total amount of H3 was used as loading control

It has been reported that IDH2 mutation damages histone demethylation and blocks cell differentiation [10–12], whereas our results showed that CP-17 can restore TF-1(IDH2/R140Q) cell differentiation. Hence, we probed whether CP-17 could change the hypermethylation of histone in TF-1(IDH2/R140Q) cells. As shown in Fig. 7, TF-1(IDH2/R140Q) cells exhibited a marked increase in the histone methylation at 3 histone marks as compared with TF-1(WT) cells, resulting in epigenetic dysregulation of differentiation block. Treatment with CP-17 (at 1 and 3 μM) for 7 days induced a dose-dependent decrease in histone methylation, thus restored the differentiation of TF-1(IDH2/R140Q) cells.

## Discussion

IDH1/2 mutations were identified in about a fifth of AML patients, contributing to leukemia by blocking differentiation of hematopoietic cells [29]. Although the recently approved drug Enasidenib exhibits encouraging therapeutic effects initially in the treatment of AML with IDH2 mutations, therapeutic resistance occurs in association with second-site IDH2 mutations after a period of treatment [21]. As a result, some patients relapse and become refractory, leading to poor prognosis. Therefore, new inhibitors of IDH2 mutants is in urgent need.

In this study, we first identified a heterocyclic urea amide compound CP-17 as an allosteric inhibitor of the mutant IDH2/R140Q enzyme by structure-based in silico screening. It was subsequently shown to inhibit the activity of IDH2/R140Q homodimer with an IC_50_ value of 40.75 nM in vitro. Notably, the inhibitory potency of CP-17 for IDH2/R140Q is 55.6 times than that for IDH2/WT, a much higher selectivity than AGI-6780 and Enasidenib. Molecular docking suggested that CP-17 binds to the allosteric site situated at the dimeric interface of IDH2/R140Q through extensive polar and hydrophobic interactions, with Q316 being the crucial interacting residue.

The enzymatic activity of IDH2/R140Q requires a closed conformation of the catalytic pocket upon the substrate binding [22, 28]. However, the binding of CP-17 leads to wider entrances of the catalytic pockets for both monomers of IDH2/R140Q than that in its absence. These results indicate that CP-17 can allosterically regulate the active sites of the enzyme and lock them in open conformations, prohibiting the catalytic conversion of α-KG to D-2-HG. Moreover, CP-17 weakens the NADPH binding with IDH2/R140Q, further inhibiting the catalytic activity of the NADPH-dependent enzyme, as negative correlation between inhibitory activities and binding free energies of NADPH was observed.

As the wild-type IDH2 plays a crucial role in human primary metabolism, the selectivity of IDH2 mutant inhibitors appear to be particularly important. CP-17 exhibits excellent selectivity with 55.6 times more potent activity against IDH2/R140Q than IDH2/WT, figuring out the mechanism of which can guide subsequent structural modification. Our MD simulation results demonstrated that CP-17 locks one monomer of the enzyme in an inactive open conformation and the other in an active closed conformation, leading to decreased enzymatic activity of IDH2/WT. Compared with IDH2/R140Q inhibited by CP-17 (both monomers are locked in open conformations), IDH2/WT is more active when treated with the same concentrations of CP-17. These results indicated that different conformation states of the catalytic pockets are closely related to the inhibited activity of IDH2 enzymes.

In vitro cellular assay results demonstrated that CP-17 inhibits intracellular D-2-HG production with an IC_50_ value of 141.4 nM and suppresses the proliferation of TF-1 erythroleukemia cells carrying the IDH2/R140Q mutant. IDH2 mutations are reported to induce a differentiation block as well as histone and DNA hypermethylation in hematopoietic cells [6, 9, 12]. CP-17 was shown to restore the EPO-induced differentiation that is blocked by IDH2/R140Q mutation and decrease hypermethylation of histone in TF-1(IDH2/R140Q) cells. Taken together, these results indicate that the selective IDH2/R140Q inhibitor CP-17 is worth for further structural optimization for the development of drug candidates against the IDH2/R140Q mutant.

## Conclusion

CP-17 identified by in silico screening was characterized as a potent inhibitor of IDH2/R140Q with IC_50_ of 40.75 nM. It exhibited excellent selectivity of over 55 folds against the wild-type IDH2. CP-17 bound at the allosteric site, locking the enzyme active sites in open conformations with abolished activity to produce D-2-HG. In vitro assays demonstrated that CP-17 could restore the EPO-induced differentiation that was blocked by R140Q mutation and decrease hypermethylation of histone in the TF-1(IDH2/R140Q) cells.

## Supplementary information

**Additional file 1.** The inhibitory activity of CP-17 and AGI-6780 against IDH2/R140Q and IDH2/WT. A. The inhibitory activity of CP-17 against IDH2/R140Q at 1 hour and 16 hours incubation. B. The inhibitory activity of AGI-6780 against IDH2/R140Q at 1 hour and 16 hours incubation. C. The inhibitory activity of CP-17 and AGI-6780 against IDH2/WT at 16 hours incubation.

## Data Availability

The data generated during this study are included in this article and its supplementary information file. Further information are available from the corresponding author on reasonable request.

## Abbreviations

α-KG: α-ketoglutarate
AML: Acute myeloid leukemia
D-2-HG: (D)-2-hydroxyglutarate
EPO: Erythropoietin
GAFF: General Amber force feld
GM-CSF: Granulocyte-macrophage colony-stimulating factor
HBG: Hemoglobin γ
HTVS: High-throughput virtual screening
IDH: Isocitrate dehydrogenase
MD: Molecular dynamics
PME: Particle-mesh Ewald method
RESP: Restrained electrostatic potential
RMSD: Root-mean-square deviation
SP: Standard-precision
XP: Extra-precision
