# Supplementary material for: Identification of a selective inhibitor of IDH2/R140Q enzyme that induces cellular differentiation in leukemia cells
Source: Cell Commun Signal. 2020 Apr 3;18:55. doi: 10.1186/s12964-020-00536-7 (PMC7126369; doi:10.1186/s12964-020-00536-7)
Supplement: Supplementary file 2 — Additional file 1. The inhibitory activity of CP-17 and AGI-6780 against IDH2/R140Q and IDH2/WT. A. The inhibitory activity of CP-17 against IDH2/R140Q at 1 hour and 16 hours incubation. B. The inhibitory activity of AGI-6780 against IDH2/R140Q at 1 hour and 16 hours incubation. C. The inhibitory activity of CP-17 and AGI-6780 against IDH2/WT at 16 hours incubation. [file 12964_2020_536_MOESM2_ESM.docx]

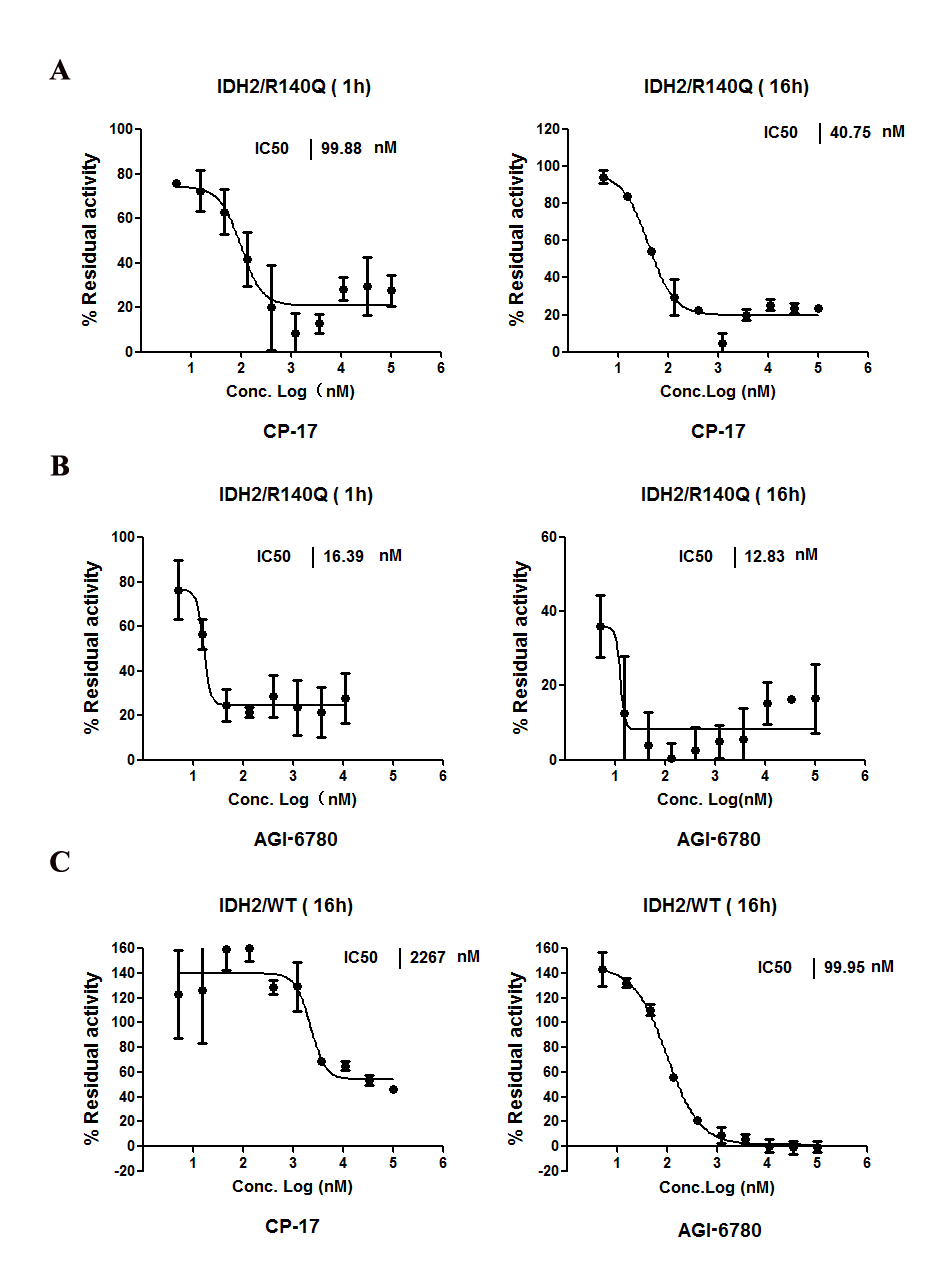


The inhibitory activity of CP-17 and AGI-6780 against IDH2/R140Q and IDH2/WT. A. The inhibitory activity of CP-17 against IDH2/R140Q at 1 hour and 16 hours incubation. B. The inhibitory activity of AGI-6780 against IDH2/R140Q at 1 hour and 16 hours incubation. C. The inhibitory activity of CP-17 and AGI-6780 against IDH2/WT at 16 hours incubation.
